# Effect of Huai Qi Huang on Epithelial-Mesenchymal Transition of Renal Tubular Epithelial Cells through miR-200a

**DOI:** 10.1155/2016/8612190

**Published:** 2016-01-14

**Authors:** Jinyun Pu, Yu Zhang, Jianhua Zhou

**Affiliations:** Department of Pediatrics, Tongji Hospital, Tongji Medical College, Huazhong University of Science and Technology, Wuhan, Hubei 430030, China

## Abstract

Epithelial-mesenchymal transition (EMT) of renal tubular epithelial cells is a vital mechanism of renal fibrosis. Mounting evidence suggests that miR-200a expression decreases in tubular epithelial cells in unilateral ureteral obstruction (UUO) rats. Moreover, it has been demonstrated that Huai Qi Huang (HQH) can ameliorate tubulointerstitial damage in adriamycin nephrosis and delay kidney dysfunction in primary glomerular disease. However, the effect of HQH on EMT of tubular epithelial cells in UUO rats and its molecular mechanism is unclear. In order to explore the effect of HQH on EMT and its molecular mechanism in renal fibrosis,* in vitro* and* in vivo* experiments were performed in our study. Our results showed that HQH increased miR-200a expression in UUO rats and in TGF-*β*1 stimulated NRK-52E cells. Meanwhile, HQH decreased ZEB1 and ZEB2 (the transcriptional repressors of E-cadherin), *α*-SMA expression in renal tubular epithelial cells* in vitro* and* in vivo*. Furthermore, we found that HQH protected kidney from fibrosis in UUO rats. The results demonstrated that HQH regulated miR-200a/ZEBs pathway and inhibited EMT process, which may be a mechanism of protecting effect on tubular cells in renal fibrosis.

## 1. Introduction

Chronic kidney disease (CKD) is considered to be an increasing public health issue, leading to end-stage renal failure. Renal tubulointerstitial fibrosis represents the most common and serious pathological changes in CKD. Besides excessive extracellular matrix accumulation, inflammatory cells infiltration, tubular epithelial cells atrophy, and myofibroblasts proliferation can also be observed in the renal interstitium [1]. It has been showed by protein tagging technology that up to 5% of myofibroblasts in UUO mouse were derived from renal tubular epithelial cells [2, 3]. Transforming growth factor *β*1 is well known to play a pivotal role in the development of renal fibrosis through regulating epithelial-mesenchymal transition of tubular epithelial cells via smad or nonsmad signal transduction pathway [4–6]. The myofibroblasts, marked with alpha-smooth muscle actin protein, appear to be the major resource of extracellular matrix in the fibrotic kidney [7]. Unilateral ureteral obstruction (UUO) is a well-established experimental model for investigating renal fibrosis [8]. However, the detailed underlying mechanism of TGF-*β*1 on epithelium-mesenchymal transition is not fully understood.

MicroRNA is one kind of short noncoding RNA molecules, which works as a negative regulator of gene expression at the posttranscriptional level. Binding to the 3′UTR sequence of target mRNAs, miRNA results in translational suppression or degradation of matched mRNAs [9]. The miR-200 family plays a vital role in inhibiting EMT induced by TGF-*β*1 in renal tubular epithelial cells. TGF-*β*1 is capable of modifying microRNAs profiles in tumor cells and normal cells [10]. Members of miR-200 family are responsible for suppressing the translation of two members of zinc-finger E-box binding homeobox family, ZEB1/*δ*EF1 and ZEB2/SIP1. It is supposed that all members of miR-200 family are able to directly target ZEB1 and ZEB2 [11, 12]. It has been demonstrated that ZEB1 and ZEB2 repress transcription of E-cadherin after combining with the enhancer box (E-box) in the E-cadherin gene [13]. However, TGF-*β*1 is showed to upregulate the transcriptional repressors ZEB1 and ZEB2 to facilitate EMT by efficiently downregulating the cell-cell adhesion molecule E-cadherin. Recent studies indicate that all members of miR-200 family, especially miR-200a, are implicated in inhibition of mesenchymal transition in tubular epithelial cells, partially mediating through E-cadherin restoration at initiation of EMT [12].

Huai Qi Huang (HQH) is mainly composed of extract out of fungus* Trametes robiniophila* Murr. (Huaier in Chinese). Huaier has been applied as a traditional Chinese medicine (TCM) for approximately 1600 years. The polysaccharide protein (PS-T) isolated from aqueous extracts of Huaier has been identified to be a major bioactive ingredient exhibiting anticancer potential and immunomodulatory actions [14]. The antitumor activities of Huaier have been widely investigated in recent years. Accumulating scientific proofs reveal that Huaier exerts antitumor effects through antiproliferation, apoptosis, and antimetastasis in cancer cell lines [15–17]. With the conduction of elaborate molecular studies, various valuable pharmacological activities of Huaier have been exploited. Huaier extract inhibits cell mobility in hepatocellular carcinoma cell MHCC97-H through downregulating N-cadherin and upregulating E-cadherin protein expressions, indicating that Huaier presents a potent potential to counteract EMT [18]. Furthermore, a recent study discovers that Huaier upregulates miR-26b-5p inducing cell apoptosis of pulmonary adenocarcinoma A549 cells [19]. Besides its antitumor activity, the plant medicine Huaier can also achieve an excellent outcome in the field of nephrology. HQH is a common prescription medicine for children with primary nephrotic syndrome (PNS) or IgA nephropathy (IgAN) in China and has shown effective in treating repeated infection, proteinuria, and hematuria [20, 21]. Several studies have found that Huaier cream or HQH granules can significantly protect podocyte against adriamycin-induced injury and ameliorate renal tubulointerstitial damage [22, 23]. Duan et al. show that elevated serum levels of creatinine, malondialdehyde (MDA), and two acute kidney injury (AKI) markers cystatin C (Cys-C) and neutrophil gelatinase-associated lipocalin (NGAL) were significantly reduced under the baseline levels by HQH, while estimated glomerular filtration (eGFR) was improved [24]. Research findings of Huaier on EMT and of HQH on renal function give us a clue that HQH might delay the progression of renal fibrosis via inhibiting renal epithelium cells EMT. In this study, we have studied the effects of HQH cream on epithelium-mesenchymal transition of renal tubular epithelial cells NRK-52E cells and renal fibrosis in UUO rats. We further showed that these therapeutic effects of HQH were probably accomplished by regulating miR-200a and ZEBs expression in cultured NRK-52E cells and UUO rat model.

## 2. Materials and Methods

### 2.1. Animals and Groups

The experiments were approved by the Tongji Hospital Animal Care and Use Committee. SPF grade male Wistar rats were housed at a constant room temperature (20 ± 2°C) and amply supplied with sterilized food and water. The rats were subjected to one-week acclimatization prior to the experiment. Totally 25 rats with mean weight of 220 ± 20 g were randomly assigned into sham operation group (*n* = 5), UUO group (*n* = 5), and the treatment group (1.5 g/kg/d, 2.25 g/kg/d and 3.0 g/kg/d; *n* = 5 in each dose group). All rats received abdominal anesthesia by 10% chloral hydrate (0.3 mL/100 g). The UUO model was established following the standard procedure [25]. After abdominal median incision, the left ureter of rats in UUO and treatment group were ligated at two places and then cut between the two ligations. The rats in sham group underwent similar surgical procedures except ureteral ligation and cut. HQH was intragastrically given to UUO rats from day 3 after obstruction at dosages of 1.5, 2.25, or 3.0 g/kg/d, respectively, while rats in the sham and UUO group were administered with normal saline. The rats in each group were sacrificed on day 14 after ligation. The left kidney was removed and the cortex was spliced into several parts; some of them were fixed in 4% paraformaldehyde for histological studies, and others were stored in liquid nitrogen for protein and RNA extractions.

### 2.2. Renal Pathological Studies

The tissue in the upper pole of the obstructed kidneys was fixed in 4% paraformaldehyde overnight and processed through dehydration and paraffin embedding. The renal tissues were cut into slices and stained with Masson's trichrome. The pathological changes were observed under the microscope on high-power fields and images were captured for semiquantitative analysis.

### 2.3. Chemicals and Reagents

The recombined human TGF-*β*1 was from PeproTech (Rocky Hill, NJ, USA), stored and used according to the instructions. The anti-E-cadherin antibody was from BD Transduction Laboratories (Franklin Lakes, NJ). The anti-*α*-smooth muscle actin antibody was from Boster company (Wuhan, China). The ChemMate EnVision Detection kit was from Dako (Carpinteria, CA, USA). The Revert Aid First Strand cDNA Synthesis Kit and SYBR Green Real-Time PCR Master Mix were obtained from Toyobo Co., LTD (Osaka, Japan). MicroRNAs were detected using the All-in-One miRNA qRT-PCR Detection Kit (GeneCopoeia, Rockville, MD, USA).

HQH cream, mainly containing aqueous extracts of* Trametes robiniophila* Murr., was brought by Gaitianli Pharmaceutical Co. LTD (Qidong, Jiangsu Province, China). For animal experiments, 15 g of HQH was dissolved in 50 mL normal saline to be a stock solution at the concentration of 0.3 g/mL and stored at 4°C after sealing. For cell experiment, 3.6 g of HQH was mixed with 20 mL complete medium to prepare a 180 mg/mL stock-solution. Then the mixture was sterilized through 0.22 filter membrane before storing at −20°C.

### 2.4. Cell Culture

Rat renal proximal tubular epithelial cell lines NRK-52E cells were obtained from the Type Culture Collection of the Chinese Academy of Sciences (Shanghai, China) and cultured in high glucose DMEM (Hyclone, USA) containing 10% fetal bovine serum (Gibco, Carlsbad, CA, USA) at 37°C with 5% CO_2_. The cells were subcultured at 80% confluence and then transferred to flasks with complete medium. The cells were stimulated with TGF-*β*1 (10 ng/mL) at gradient concentrations (60, 120, and 180 ug/mL) of HQH subsequent to starvation treatment with serum-free medium for 12 hours.

### 2.5. Cell Immunofluorescence Staining

NRK-52E cells were subcultured on cover slips. At the end point, the slips were washed twice in precooling PBS and then fixed in 4% paraformaldehyde for 10 minutes. After washing twice, the fixed cells were permeated with 0.1% TritonX-100 for 5 min, and then the cells were rinsed in PBS twice for 5 min. The cells were blocked with 5% BSA and incubated with specific primary antibodies at 4°C overnight. After washing sufficiently with PBS, the cells were incubated with FITC-conjugated secondary antibody for 60 minutes. Slides were viewed with Olympus optical fluorescence microscope. The immunofluorescent images were photographed with equal exposure settings.

### 2.6. Immunohistochemical Studies

After deparaffinization, the 3 *μ*m sections of renal tissues were hydrated and immersed in methanol containing 3% H_2_O_2_ in the dark for 30 min. And then, they were incubated with epitope retrieval solution at high pressure (103.4 kPa) for 3 min, and thereafter with 5% bovine serum albumin at 37°C for 60 min to block nonspecific binding. The sections were incubated with anti-*α*-SMA primary antibodies (1 : 400) or anti-E-cadherin primary antibodies (1 : 800), respectively, overnight at 4°C. After rinsing in 0.01 M PBS three times, the sections were performed with ChemMate EnVision Detection kit at room temperature according to the instructions. Ten nonoverlapping fields of sections were captured and the positive areas in each field were measured using Image Pro Plus software.

### 2.7. Western Blot Analysis

Total proteins were extracted from renal cortex tissues or cells in RIPA with PMSF on ice for 30 min; then the lysate was centrifuged at 4°C to collect supernatant for the experiments. The protein concentration of the supernatant was determined by Bradford assay and the supernatant was mixed with SDS loading buffer. The sample mixtures were boiled for 5 minutes to denature protein and then were electrophorised in SDS polyacrylamide gel and then transferred onto PVDF membranes. The nonspecific background bindings were blocked in 5% BSA for 1 h at room temperature; then the immunoblots were incubated overnight with antibodies anti-E-cadherin (1 : 2000), anti-*α*SMA (1 : 500), and anti GAPDH (1 : 10000) at 4°C. After several washes in TBS-Tween 20, the membranes were incubated with HRP-conjugated secondary antibody (1 : 10000) for 1 h at room temperature. The bands of specific protein were detected by ECL detection kits. The optical density of bands was quantified with GAPDH as an internal inference.

### 2.8. RT-qPCR

Total RNA from the renal tissue was extracted using the HiPure Universal RNA Kits according to the manufacturer's manual. First-strand cDNA was synthesized according to the instructions. The primers were blasted in NCBI Genebank, and primers for ZEB1, ZEB2, and *β*-actin were listed as the following: *β*-actin sense, 5′-CAG CTG AGA GGG AAA TCG TG-3′ and anti-sense, 5′-CGT TGC CAA TAG TGA TGA CC-3′; ZEB1 sense, 5′-TGG GAA AGC GTT CAA GTA CAA A-3′ and anti-sense, 5′-TTG GTT TAC AGA AAG CGG TTC TT-3′; ZEB2 sense, 5′-AGC CAA GGA ATG CTA CCA A-3′ and anti-sense, 5′-GGC CCC AGA GCA TCA TAA TC-3′. Real-time PCR was performed with a Roche real-time quantitative PCR detection system using SYBR Green PCR Master Mix following the manufacturer's manual. PCR amplifications were performed under the following reaction conditions: 95°C for 2 min; 40 cycles at 95°C for 15 s, 60°C for 30 s, and 72°C for 30 s. The miRNA-200a was quantitatively detected with All-in-One miRNA qPCR detection Kit according to the manual. The results were expressed relative to *β*-actin or U6, respectively. The relative expression amounts were analyzed using the equation 2^−ΔΔCt^, in which ΔΔCt = ΔCt_treatment_ − ΔCt_control_.

### 2.9. Statistical Analysis

All the data in figures were expressed as the mean ± SEM. One-way ANOVA statistical analysis was performed and the statistical significance between groups was evaluated by a LSD test. The *p* value under 0.05 indicates statistical significant difference using SPSS 13.0 software.

## 3. Results

### 3.1. HQH Inhibited TGF-*β*1-Induced EMT in NRK-52E Cells

**Figure 1:**
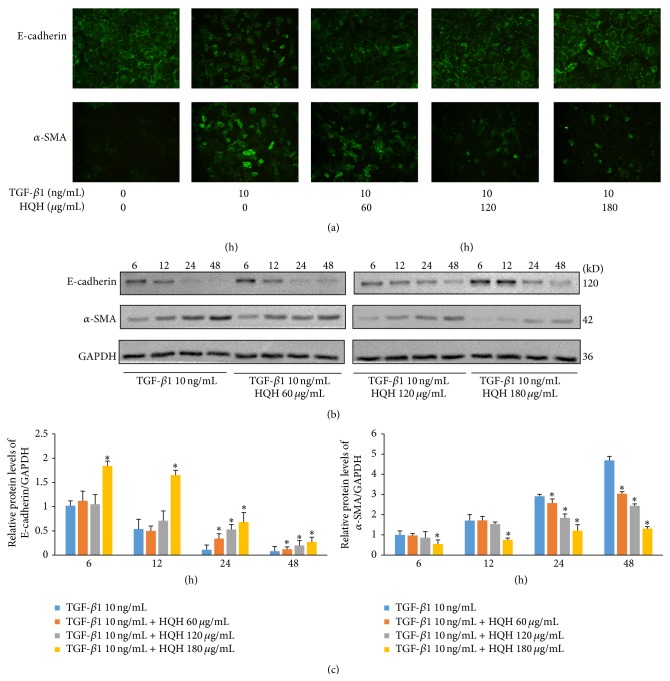
Effects of Huai Qi Huang (HQH) on E-cadherin and *α*-SMA protein expression in NRK-52E cells stimulated with transforming growth factor-*β*1 (TGF-*β*1). (a) TGF-*β*1 induces downregulated expression of E-cadherin but upregulated expression of *α*-SMA in NRK-52E cells; HQH displays inhibitory effect on phenotypic changes and protein expression of E-cadherin and *α*-SMA in NRK-52E cells at 24 h (immunofluorescence, 200x). (b and c) Western blot and quantitative analysis of E-cadherin and *α*-SMA protein expression in NRK-52E cells in each group at 6 h, 12 h, 24 h, and 48 h. ^*∗*^ *p* < 0.05 versus the TGF-*β*1 group at the same time point. All experiments were repeated for three times. The bars show mean ± SEM.

Treatment of NRK-52E cells with 10 ng/mL TGF-*β*1 for 24 hours showed downregulated expression of E-cadherin but upregulated expression of *α*-SMA. HQH at gradient concentrations (60 ug/mL, 120 ug/mL, and 180 ug/mL) could significantly counteract against the transition induced by TGF-*β*1 (Figure 1(a)). As shown in Western blot and quantitative analysis (Figures 1(b) and 1(c)), HQH increased E-cadherin and decreased *α*-SMA in time and dose-dependent manner, which indicates that HQH can directly suppress TGF-*β*1 induced EMT in NRK-52E cells.

### 3.2. HQH Downregulated Zinc Finger E-Box-Binding Homeobox

**Figure 2:**
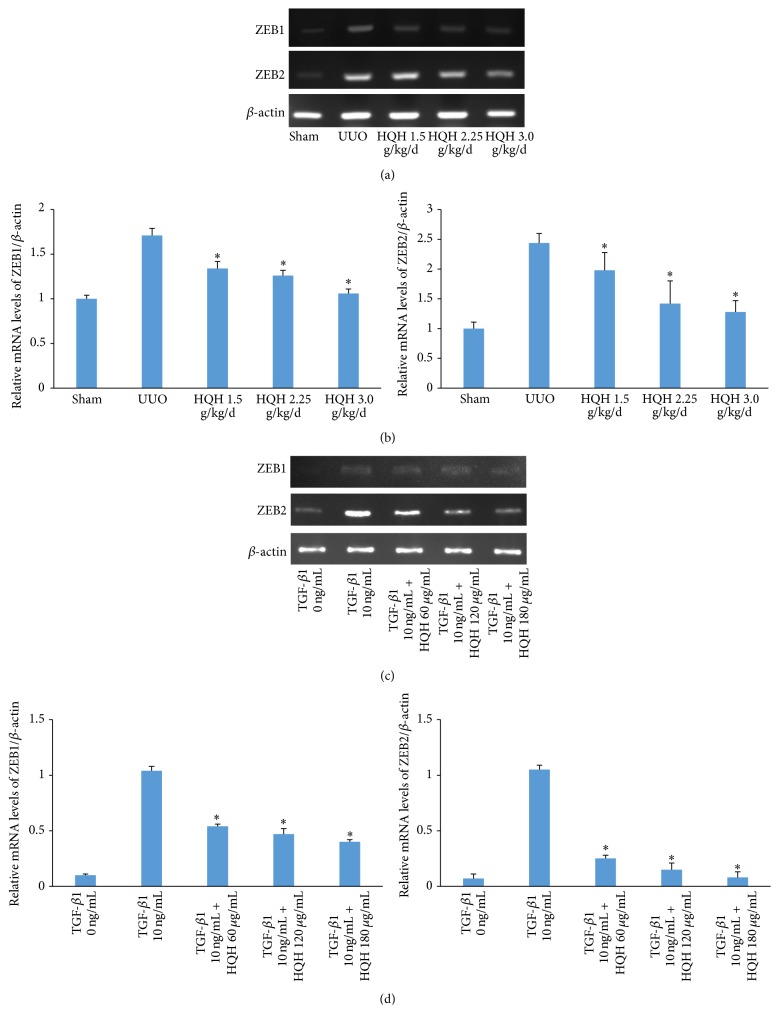
Effects of Huai Qi Huang (HQH) on zinc-finger E-box binding homeobox ZEB1 and ZEB2 mRNA levels in the obstructed kidneys of UUO rats and NRK-52E cells stimulated with transforming growth factor-*β*1 (TGF-*β*1). (a and b) Representative RT-PCR bands and graphic presentation showed levels of ZEB1 and ZEB2 mRNA in obstructed kidneys on day 14 after obstruction. ^*∗*^ *p* < 0.05 versus vehicle-treated UUO rats on the same day after obstruction. Five rats in each dosage group. The bars show mean ± SEM. (c and d) The relative abundance of ZEB1 and ZEB2 mRNA induced by 10 ng/mL TGF-*β*1 treatment for 3 h in NRK-52E cells. ^*∗*^ *p* < 0.05 versus positive control group with the TGF-*β*1 group at the same time point. All experiments were repeated for three times. The bars showed mean ± SEM.

To explore the potential mechanisms involved in EMT inhibition by HQH, we further investigated ZEB1 and ZEB2 gene expression using RT-qPCR in obstructed kidneys of UUO rats and TGF-*β*1 stimulated NRK-52E cells. ZEB1 mRNA expression was almost undetectable and ZEB2 mRNA expression was very low in sham operation rats, but their expression were significantly increased in obstructed kidneys on day 14. UUO rats treated with HQH showed a significant decline in ZEBs mRNA expression on day 14 after obstruction (Figures 2(a) and 2(b)). This is consistent with results* in vivo*. HQH also dramatically inhibited ZEBs mRNA expression in TGF-*β*1-treated NRK-52E cells at 3 h (Figures 2(c) and 2(d)). Consequently, it seems clear that HQH might be an effective drug to inhibit ZEBs mRNA levels in obstructed kidney and in NRK-52E cells stimulated with TGF-*β*1.

### 3.3. HQH Upregulated miRNA-200a Expression in UUO Rats and NRK-52E Cells

**Figure 3:**
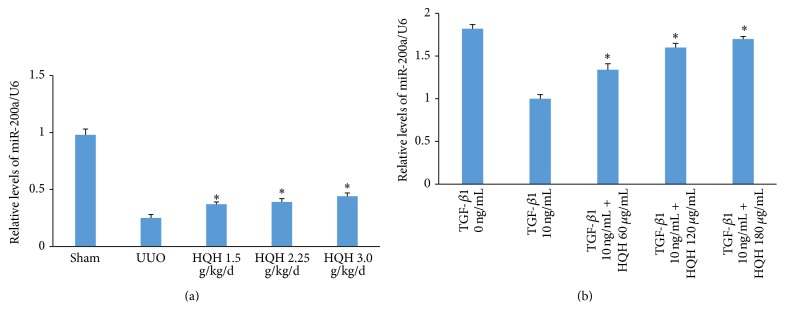
Effect of Huai Qi Huang (HQH) on the expression level of miRNA-200a in UUO rats and in transforming growth factor-*β*1- (TGF-*β*1-) treated NRK-52E cells. (a) The relative abundance of miR-200a in obstructed kidneys of UUO rats on day 14. ^*∗*^ *p* < 0.05 versus vehicle-treated UUO rats on day 14 after obstruction. Five rats in each dosage group. The bars show mean ± SEM. (b) The relative abundance of miR-200a in NRK-52E cells stimulated with TGF-*β*1 and HQH. ^*∗*^ *p* < 0.05 versus positive control group with the TGF-*β*1 group at the same time point.

The miR-200a expression was examined in obstructed kidneys of UUO rats in each group by quantitative real-time PCR. The miR-200a expression was significantly downregulated by three quarters in obstructed kidney in UUO rats compared to sham operation rats on day 14. Different dosages of HQH upregulated miRNA-200a expression after obstruction (Figure 3(a)). The miR-200a was downregulated at 3 h in NRK-52E cells stimulated with TGF-*β*1. HQH at concentration of 60, 120, and 180 ug/mL restored miR-200a expression in TGF-*β*1-stimulated NRK-52E cells (Figure 3(b)). It indicated that HQH sustained miRNA-200a levels during EMT in renal fibrosis.

### 3.4. Effects of HQH on Epithelial-Mesenchymal Transition in UUO Rats

**Figure 4:**
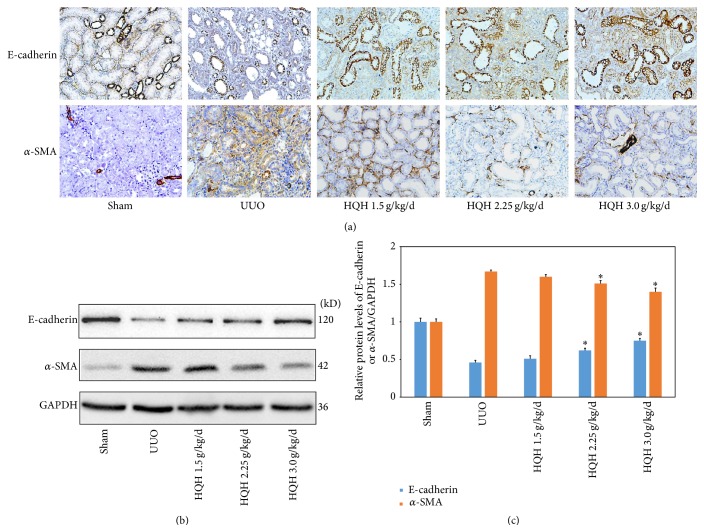
Huai Qi Huang (HQH) inhibited epithelial-mesenchymal transition (EMT) in UUO rats. (a) Immunohistochemical staining shows E-cadherin^+^ epithelial cells and *α*-SMA^+^ myofibroblasts distributed in sham operation and obstructed kidney in each group (200x). (b) Representative bands for E-cadherin and *α*-SMA protein detected by Western blots in obstructed kidneys of UUO rats. (c) Relative protein levels of E-cadherin or *α*-SMA to GAPDH in each group. ^*∗*^ *p* < 0.05 versus vehicle-treated UUO rats on the same day after obstruction. Five rats in each dosage group. The bars show mean ± SEM.

To understand a possible mechanisms of HQH to counteract renal fibrosis, we detected E-cadherin and *α*-SMA protein expression in kidneys using IHC and Western blot assay. In sham rats, E-cadherin, as a cell adhesion molecule, was highly expressed in renal tubular epithelial cells, while *α*-SMA was expressed mainly in vascular smooth muscle cells. In UUO rats, part of the renal tubular epithelial cells lost E-cadherin with clear increase of *α*-SMA positive cells distributed around injured tubules. HQH partly impacted on E-cadherin distribution of renal tubular epithelial cells in UUO rats. In addition, the density of *α*-SMA positive cells accumulated in renal interstitium decreased significantly in UUO rats with HQH treatment (Figure 4(a)). In sham rats, E-cadherin protein was highly expressed, while little *α*-SMA protein was detected. In UUO rats, E-cadherin was lost with significant increase of *α*-SMA. HQH partly impacted on E-cadherin distribution of renal tubular epithelial cells in UUO rats (Figure 4(b)). In accordance with the above results, quantitative analysis data of protein bands showed that HQH restored E-cadherin protein and reduced *α*-SMA protein in UUO rats (Figure 4(c)).

### 3.5. HQH Attenuated Renal Fibrosis in UUO Rats

**Figure 5:**
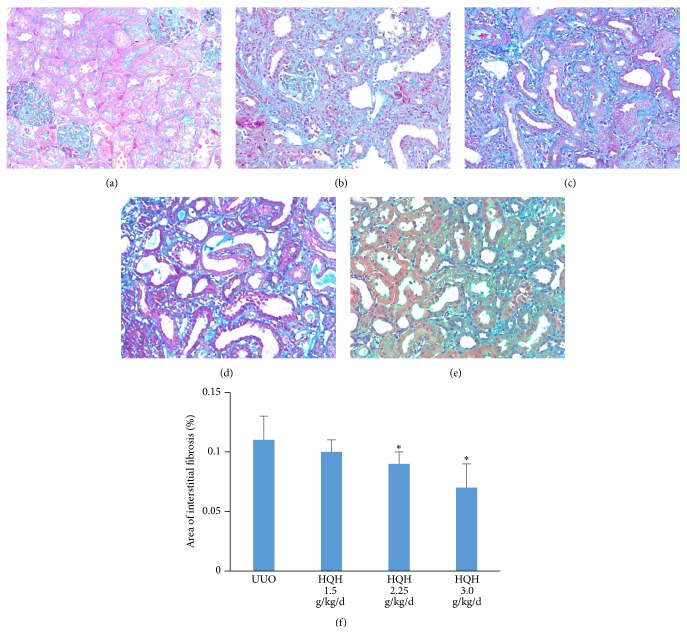
Effects of Huai Qi Huang (HQH) on renal interstitial fibrosis in UUO rats on day 14 (Masson's staining, 200x). (a) Sham rats. (b) UUO rats. (c) HQH 1.5 g/kg/d treatment group. (d) HQH 2.25 g/kg/d treatment group. (e) HQH 3.0 g/kg/d treatment group. (f) Area of interstitial fibrosis in each group. ^*∗*^ *p* < 0.05 versus vehicle-treated UUO rats on the same day after obstruction. Five rats in each dosage group. The bars indicate mean ± SEM.

To observe the efficacy of HQH to delay the progress of renal fibrosis, we started HQH treatment from day 3 after obstruction. Fibrous deposition in interstitium was observed by visualization staining. There were severe renal fibrosis companied by diffuse degeneration in tubular epithelial cells, dilation or atrophy in most tubules, and multifocal infiltration of inflammatory cells on day 14 (Figure 5(b)). HQH treatment appeared to be effective in reducing renal fibrosis when compared to the untreated UUO rats (Figures 5(c)–5(e)), especially in groups with higher dosages at 2.25 and 3.0 g/kg/d (Figure 5(f)). The treatment with HQH could significantly attenuate renal fibrosis in UUO rats at dosage-dependent manner.

## 4. Discussion

The present study demonstrated that administration of Huai Qi Huang (HQH) cream could slow down the progression of renal fibrosis in unilateral ureteral obstructive rats* in vivo*. Furthermore, it showed that HQH inhibited TGF-*β*1-induced EMT in NRK-52E cells directly* in vitro*. The potential mechanism of its antifibrotic effect is involved in EMT inhibition by regulating miR-200a/ZEBs signaling in renal tubular epithelial cells. Our data showed that administration of HQH could significantly ameliorate renal fibrosis by inhibiting tubular EMT process.

Renal tubular injuries lead to rapidly progressive renal interstitial fibrosis in unilateral ureteral obstruction rat model, resulting from mechanical stress and osmotic pressure with various molecular mediators [26]. As early as day 7 after obstruction, histopathological lesions are observed, such as tubular atrophy, inflammation cells infiltration, and ECM deposition. Renal fibrosis results mainly from excessive accumulation of myofibroblast, which is capable of helping repair damaged tissue through producing extracellular matrix and synthesizing *α*-SMA to generate contractile tension [26]. However, origin of myofibroblasts in renal fibrosis has been on debate. Iwano et al. discovered that more than 30% of fibroblasts derive from proximal tubular cells via EMT [3]. However, the investigation by Lebleu et al. shows that only around 5% of the myofibroblast population is tracked from renal epithelial cells [27]. Grande et al. discover that tubular epithelial cells fail to delaminate across the basement membrane to be members of myofibroblast population but just undergo partial EMT after renal injury [28]. Damaged tubular epithelial cells undergo EMT and then transfer crucial signals towards renal interstitium to activate phenotypic transition of fibroblasts and sustain inflammation, resulting in progressive renal fibrosis [29, 30]. These studies indicate that EMT is an important event during renal fibrosis, is of functional outcomes to kidney abnormalities, and might be targeted to inhibit renal fibrosis. HQH treatment sustains E-cadherin in renal epithelial cells and decreases *α*-SMA positive cells simultaneously. HQH was able to inhibit EMT by reversing the increase of *α*-SMA and decrease of E-cadherin expression in UUO rats, indicating its antifibrotic effect. Further* in vitro* study showed that HQH had direct inhibitory effect on EMT in NRK-52E cells induced by TGF-*β*1. However, our results* in vitro* showed that lower dosages of HQH are insufficient to take effect on EMT at 6–12 h. It indicates that HQH inhibits EMT in dose- and time-dependent way. These data indicate that HQH has effects on EMT to produce a significant diminishment in myofibroblasts accumulation during renal fibrosis.

Although many factors are related to EMT in renal fibrosis, the underlying molecular mechanism for the occurrence and development of this complicated course has not been fully clarified. Reduction of E-cadherin during EMT is induced by ZEB1/ZEB2 through repressing E-cadherin genes transcription in epithelial cells. Emerging evidences revealed that miR-200 plays a vital role in maintaining epithelial phenotype. Recent studies have linked up miR-200 members to ZEBs [31] and revealed that miR-200 family mainly regulates EMT through directly targeting ZEB1 and ZEB2 mRNA. It had been demonstrated that ZEBs were implicated in early period of tubular EMT [12]. Downregulation of miR-200 family was found significantly on day 7 after obstruction and more obviously on day 14 in UUO rats, which is accountable for initiation of EMT in tubular epithelial cells during renal fibrosis [12, 32]. The present data showed that HQH could restore the downregulated expression of miR-200a in UUO rats and reduced ZEBs expression at 3 h and inhibited EMT at 24 h in NRK-52E cells. The results indicate that HQH inhibited renal fibrosis and EMT probably by regulating miR-200a/ZEBs expression.

## 5. Conclusion

In summary, our data demonstrate that HQH can effectively inhibit renal fibrosis in UUO rats and EMT of renal tubular cells probably by regulating miR-200a/ZEBs expression. Our results contribute to the knowledge regarding HQH treatment in renal disease treatment. However, the detailed molecular mechanisms of HQH treatment to renal fibrosis require advanced exploration in following studies.
